# Teriparatide Treatment for Hypercalcemia Associated With Adynamic Bone Disease

**DOI:** 10.1002/jbm4.10176

**Published:** 2019-02-27

**Authors:** Jennifer Peugh, Andrew Khalil, Micah R Chan, Karen E Hansen

**Affiliations:** ^1^ Internal Medicine University of Wisconsin School of Medicine and Public Health Madison WI USA; ^2^ Department of Orthopedics and Rehabilitation University of Wisconsin School of Medicine and Public Health Madison WI USA; ^3^ Nephrology Division Department of Medicine University of Wisconsin School of Medicine and Public Health Madison WI USA; ^4^ Rheumatology Division Department of Medicine University of Wisconsin School of Medicine and Public Health Madison WI USA

**Keywords:** HYPERCALCEMIA, TERIPARATIDE, ADYNAMIC BONE DISEASE, CKD‐MBD, DIALYSIS

## Abstract

Hypercalcemia most often results from primary hyperparathyroidism and malignancy. Adynamic bone disease (ABD) is a form of renal osteodystrophy characterized by reduced bone turnover, which can limit the ability of bone to release or store calcium, potentially leading to low, normal, or high serum calcium levels. We describe a 51‐year‐old dialysis‐dependent female with hypercalcemia after parathyroidectomy. A demeclocycline‐labeled bone biopsy confirmed adynamic bone disease. Teriparatide, a recombinant form of parathyroid hormone (PTH) used to treat postmenopausal osteoporosis, was prescribed for 12 months and normalized serum calcium levels. Although previous case reports and series have described favorable changes in spine bone mineral density when teriparatide was prescribed for ABD, ours is the first documented case in which teriparatide resolved hypercalcemia due to ABD. © 2019 The Authors. *JBMR Plus* published by Wiley Periodicals, Inc. on behalf of American Society for Bone and Mineral Research.

## Introduction

Humans achieve calcium homeostasis through an intricate balance between multiple organ systems including the kidneys, bones, gastrointestinal tract, and parathyroid glands. Although hypercalcemia most often results from either primary hyperparathyroidism or malignancy, any disease of these organs may lead to dysregulation of calcium balance and variations in serum calcium.^1^ Parathyroid hormone (PTH) tightly regulates serum calcium through multiple mechanisms: PTH increases renal 1,25(OH)_2_D synthesis to promote intestinal calcium absorption, enhances renal reabsorption of calcium in the distal tubules, and increases bone resorption to maintain normocalcemia. Healthy individuals achieve neutral calcium balance via adequate calcium ingestion, intestinal absorption, and renal reabsorption, which together support normal mineralization of bone.^2^

Chronic kidney disease (CKD) allows for multiple sites of dysregulated calcium homeostasis.^3^ Decreased renal 1,25(OH)_2_D synthesis reduces intestinal calcium absorption and renal calcium reabsorption. Hypocalcemia stimulates parathyroid gland release of PTH, which upregulates renal 1,25(OH)D_2_D synthesis and osteoclastic bone resorption to release skeletal calcium. However, when PTH levels are chronically elevated, CKD patients can develop skeletal resistance to PTH. Thus, CKD often causes negative calcium balance. On the other hand, prescription of calcium‐based phosphate binders, vitamin D supplements, or calcimimetics can maintain neutral or positive calcium balance.^3^ Additionally, the dialysate calcium concentration can promote positive calcium balance. Because 99% of total body calcium is stored in the skeleton and only 0.025% of calcium is found in the serum, a severe imbalance in total body calcium may exist, with only minor changes in the serum calcium.^4^

Here, we present a 51‐year‐old woman on dialysis with persistent hypercalcemia despite low calcium intake and no vitamin D supplements. A bone biopsy led to the correct diagnosis and knowledge of pathophysiology permitted successful treatment of hypercalcemia.

## Case Summary

A 51‐year‐old white female had systemic lupus erythematosus (SLE) complicated by lupus nephritis, stage CKD 5 on dialysis, secondary hyperparathyroidism prompting subtotal parathyroidectomy, hypertension, hypothyroidism, Wolf‐Parkinson‐White Syndrome, and gastroesophageal reflux disease. SLE was diagnosed in 1986 and characterized by skin, joint, and renal involvement with elevated antinuclear (titer 1:1280), dsDNA and SSA antibodies. In 1989, she developed lupus nephritis treated with prednisone and 6 months of cyclophosphamide. She took hydroxychloroquine from 1989 until 1995 after which she remained off all pharmacotherapy for SLE.

Her renal disease progressed and she began peritoneal dialysis in March 2011. At this time, she was diagnosed with secondary hyperparathyroidism (serum PTH 1382 pg/mL, normal 14–72 pg/mL; serum calcium corrected for albumin, 10.9 mg/dL, and phosphorous 6.8 mg/dL). In August 2011, she underwent 3.5 gland parathyroidectomy. Intraoperative PTH values dropped from 1257 pg/mL to 195 pg/mL. The three removed parathyroid glands weighed 0.4 g, 0.13 g, and 0.12 g. Pathology revealed hypercellular parathyroid tissue. After surgery, she took calcitriol 0.25 mcg daily and calcium carbonate 2000 mg four times daily. She continued a multivitamin, which she was also taking before surgery, containing ascorbic acid, thiamine, riboflavin, niacinamide, pyridoxine, folate, cobalamin, biotin, and pantothenic acid once daily. Her phosphate binder switched from calcium acetate with sevelamer carbonate to lanthanum carbonate during this time to control hyperphosphatemia.

**Figure 1:**
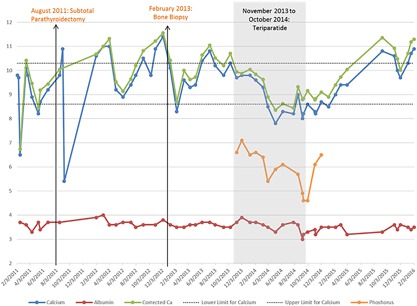
Normalization of serum calcium and phosphorus levels with use of teriparatide for adynamic bone disease.

The patient developed severe fluctuations in serum calcium levels after parathyroidectomy. One month after parathyroidectomy, symptomatic hypocalcemia required intravenous calcium (Fig. 1). Over the next 15 months, she developed asymptomatic hypercalcemia and hyperphosphatemia that persisted despite stopping calcitriol and calcium supplements (Fig. 1) and changing to a low‐calcium dialysate. Between November 2005 and September 2012, she experienced a 16% increase in L_1_ to L_4_ bone mineral density (BMD), a 16% increase in mean total hip BMD, and a 27% decrease in the 33% radius BMD. Her femur *T*‐score of −2.9 indicated “osteoporosis.”^5^ Fortunately, a vertebral fracture assessment study demonstrated no grade 2 or 3 fractures.

**Figure 2:**
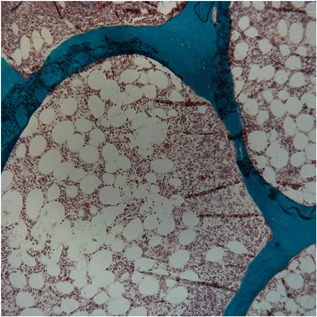
Demeclocycline‐labeled trans‐iliac bone biopsy with Goldner's stain demonstrating adynamic bone disease characterized by low bone formation with normal mineralization and bone volume. Magnification ×20.

The patient was referred to the University of Wisconsin Osteoporosis Clinic for a demeclocycline‐labeled bone biopsy to clarify the cause of her persistent hypercalcemia. She completed a 7‐day food diary indicating that she consumed 596 mg calcium/day. Her serum calcium corrected for albumin was 10.6 mg/dL (normal, 8.5–10.2 mg/dL), phosphorus was 6.4 mg/dL (2.5–4.5 mg/dL), PTH was <2 pg/mL (14–72 pg/mL), 25(OH)D was 21 ng/mL (30–80 ng/mL), 1,25(OH)_2_D was 22 pg/mL (15–75 pg/mL), and bone‐specific alkaline phosphatase was 8.8 μg/L (7–22.4 μg/L). A bone biopsy was obtained from the anterior iliac crest. The bone biopsy (Fig. 2) showed a low bone formation rate, normalized for volume and for surface. Mineralization was normal based on osteoid thickness and mineralization lag time. Cancellous bone volume was normal. The combination of low bone turnover, normal mineralization, and normal bone volume confirmed a diagnosis of adynamic bone disease (ABD).

Based on the mechanism by which the patient developed ABD (parathyroidectomy) and the knowledge that teriparatide increases bone turnover, we hypothesized that teriparatide would lower the patient's serum calcium and phosphorus levels. Treatment was delayed because her insurance plan would not cover the drug. However, ultimately, the patient began teriparatide as 20 μg subcutaneous thrice weekly, increasing within 1 month to 20 μg daily. The rationale behind starting a lower dose of teriparatide was twofold: to reduce the severity of bone pain that our other ABD patients often report when starting teriparatide and to ensure that serum calcium levels did not further increase during therapy. The drug was well tolerated and initially used for 12 months, during which laboratory studies were closely monitored (Fig. 1). Calcium levels decreased into the normal range while taking teriparatide and phosphorus levels also declined. One year after stopping teriparatide, the patient had recurrent hypercalcemia and resumed teriparatide; her serum calcium levels quickly normalized.

## Discussion

Mineral and bone disorders (CKD‐MBD), formerly known as renal osteodystrophy, are a common complication of CKD. CKD‐MBD includes osteitis fibrosa cystica, osteomalacia, ABD and a mixed subtype. These diseases are distinguished from one another by microscopic grading of bone turnover (high, normal, low), mineralization (normal or abnormal), and bone volume (high, normal, low).^6^

ABD is a common form of CKD‐MBD among the dialysis population. One series found ABD in nearly 60% of 630 dialysis patients,^7^ while another reported ABD in 19% of patients receiving hemodialysis and 50% of patients receiving peritoneal dialysis.^8^ ABD is characterized by low turnover, normal mineralization, and low to normal bone volume. Bone biopsies of patients with ABD show few osteoblasts and osteoclasts, decreased bone formation, and diminished activation frequency. Most patients with ABD have normal or low serum calcium levels, but hypercalcemia occasionally occurs due to low bone formation with inability to store calcium in the skeleton reservoir.^4^ ABD increases the risk of fractures and can promote soft tissue and vascular calcification with subsequent vascular events.^9^, ^10^

ABD results from parathyroidectomy or oversuppression of PTH.^9^, ^10^, ^11^ Beyond parathyroidectomy, risk factors for ABD include peritoneal dialysis, glucocorticoid and bisphosphonate use, diabetes, menopause, hypogonadism, increasing age, malnutrition, systemic inflammation, calcimimetics, excessive vitamin D and/or calcium supplementation, high calcium dialysate, and calcium‐based phosphate binders.^9^, ^10^

ADB can be challenging to diagnose without a bone biopsy. The diagnostic performance of serum intact PTH, whole PTH, and bone‐specific alkaline phosphatase for the diagnosis of CKD‐MBD was evaluated in nearly 500 dialysis patients who underwent bone biopsies and had stored serum available for analysis.^12^ Stored serum was analyzed at a single reference laboratory. None of the three laboratory tests showed high sensitivity and specificity, but intact PTH performed “best” of the three. A single intact PTH <2 times the upper limit of normal was 66% sensitive and 65% specific for ABD.^12^ KDIGO guidelines recommend using sequential, rather than single, PTH tests to diagnose CKD‐MDB. Although a persistently low PTH can suggest ABD, bone biopsy remains the gold standard method to confirm ABD.^13^

The diagnosis of ABD becomes even more challenging in patients taking biotin, which can falsely lower the measured PTH.^14^ Of note, our patient was taking biotin when her PTH was >1000 pg/mL and also when her PTH was undetectable. Biotin's half‐life is between 2 and 22 hours; patients with normal renal function should stop taking biotin for at least 24 hours before measurement of PTH.^14^ Biotin is also removed during hemodialysis. In one study, dialysis patients consuming biotin 1 mg daily had high serum biotin levels before dialysis, but after dialysis their biotin levels were similar to those of healthy individuals taking no biotin.^15^ In dialysis patients, PTH should be measured just after dialysis, or after biotin has been discontinued.

Treatment for ABD focuses on stimulating bone turnover by minimizing calcium intake, stopping calcium‐based phosphate binders, decreasing dialysate calcium, and avoiding calcimimetics, bisphosphonates, and vitamin D. It has been hypothesized that teriparatide would be effective for ABD based on its ability to promote both osteoblast and osteoclast activity.^16^ Because teriparatide promotes bone formation, it should also allow the skeleton to recover its function as a reservoir for excess calcium and phosphorus. For that reason, we trialed teriparatide in our patient and observed normalization of her serum calcium and phosphorus levels.

We reviewed the literature with assistance from an academic librarian to identify and summarize prior reports using teriparatide to treat ABD. We found four case reports and three case series reflecting treatment of 30 patients, summarized in Table 1. The case reports noted increased bone turnover and bone volume or BMD after 8 to 24 months of teriparatide given to 4 patients with biopsy‐proven ABD.^17^, ^18^, ^19^, ^20^ Cejka and colleagues^11^ administered teriparatide for ≤6 months to 7 CKD patients with ABD based on PTH <100 pg/mL (*n* = 6) or bone biopsy (*n* = 1). Teriparatide significantly increased lumbar spine BMD without altering femoral neck BMD, bone turnover markers, or coronary artery calcification. Cejka and colleagues also observed a significant decline in serum phosphorus (5.52 ± 0.75 to 4.23 ± 0.98 mg/dL, *p* = 0.04) without changes in serum calcium levels after use of teriparatide.^11^ Mitsopoulos and colleagues^21^ prescribed teriparatide 20 μg thrice weekly for 16 months to 8 dialysis patients with biopsy‐proven ABD and detected no significant improvement in spine or hip BMD. Samida and colleagues^22^ administered teriparatide 56.5 μg subcutaneous once weekly to 22 dialysis‐dependent patients with serum PTH <60 pg/mL, many of whom had undergone prior parathyroidectomy. Nearly half of subjects discontinued teriparatide because of adverse side effects such as transient hypotension. Among 11 subjects who completed 48 weeks of therapy with paired bone density tests at baseline and 48 weeks, spine BMD increased by 3% ± 1.8% (*p* < 0.05) without significant changes in femoral neck or 33% radius BMD. None of the 30 patients noted above had hypercalcemia as a manifestation of ABD.

**Table 1:** Summary of Studies Using Teriparatide to Treat Adynamic Bone Disease

Author, year	Bone biopsy confirmation	Intervention	Outcome	Results
Lehmann, 2009	1 of 1 patient	Teriparatide 20 μg SQ daily for 8 months	Change in bone histology	Increase in mineralized bone volume and bone turnover
Cejka, 2010	1 of 7 patients	Teriparatide 20 μg SQ daily for 6 months plus calcium and/or calcitriol	Change in BMD and CAC	Significant increase in spine but not femoral neck BMD; no change in CAC (n = 6)
Mitsopoulos, 2012	8 of 8 patients	Teriparatide 20 μg SQ thrice weekly for ∼16 months	Change in BMD	No significant change in spine or femoral neck BMD
Giamalis, 2015	1 of 1 patient	Teriparatide 20 μg SQ + cholecalciferol 400 IU + alphacalcidol 0.25 μg daily + sevemelar daily	Clinical improvements	Increase in bone turnover and BMD, improved mobility and reduced pain
Palcu, 2015	1 of 1 patient	Teriparatide 20 μg SQ daily for 24 months	Clinical improvements	Increase in bone turnover and BMD, improved mobility and reduced pain
Sumida, 2016	0 of 11 patients	Teriparatide 56.5 μg once a week for 48 weeks	Change in BMD and serum bone turnover markers	11 completers had increased bone turnover and spine BMD
Fahrleitner‐Pammer, 2017	1 of 1 patient	Teriparatide 20 μg SQ daily for 12 months	Clinical improvements	Increased bone turnover and volume, stable BMD

BMD = bone mineral density; CAC = coronary artery calcification score.

Herein, we describe a patient on peritoneal dialysis who developed secondary hyperparathyroidism and underwent subtotal parathyroidectomy. Both peritoneal dialysis and parathyroidectomy were risk factors for her subsequent ABD.^8^, ^9^, ^10^ She subsequently developed hypercalcemia and hyperphosphatemia with a low PTH; however, the concomitant use of biotin could have falsely lowered her PTH level. ABD was confirmed by trans‐iliac bone biopsy. Teriparatide successfully lowered her serum calcium and phosphorus levels, consistent with the physiologic effects of intermittent PTH stimulation to promote anabolic bone formation, allowing excess calcium and phosphorus to deposit into the skeletal reservoir.

Ours is the first case report describing use of teriparatide to treat hypercalcemia from ABD. Further research is needed to evaluate the effect of teriparatide on clinical outcomes in patients with ABD, including optimal dose, duration, and long‐term effects on BMD, fractures, soft tissue/vascular calcification, vascular events, and all‐cause mortality.

Take‐home points from this patient's story include the following:

- Parathyroidectomy and peritoneal dialysis are risk factors for ABD.
- ABD can cause low, normal, or high serum calcium levels.
- Biotin supplements can falsely lower the measured PTH.
- Teriparatide can be used to treat hypercalcemia resulting from ABD.

## Disclosures

All authors state that they have no conflicts of interest.
